# Investigating paranormal phenomena: Functional brain imaging of telepathy

**DOI:** 10.4103/0973-6131.43543

**Published:** 2008

**Authors:** Ganesan Venkatasubramanian, Peruvumba N Jayakumar, Hongasandra R Nagendra, Dindagur Nagaraja, R Deeptha, Bangalore N Gangadhar

**Affiliations:** National Institute of Mental Health and Neurosciences, Bangalore, India; 1 Swami Vivekananda Yoga Anusandhana Samsthana, Vivekananda Yoga Research Foundation, Bangalore, India

**Keywords:** fMRI, parahippocampal gyrus, telepathy

## Abstract

**Aim:**

“Telepathy” is defined as “the communication of impressions of any kind from one mind to another, independently of the recognized channels of sense”. Meta-analyses of “ganzfield” studies as well as “card-guessing task” studies provide compelling evidence for the existence of telepathic phenomena. The aim of this study was to elucidate the neural basis of telepathy by examining an individual with this special ability.

**Materials and Methods:**

Using functional MRI, we examined a famous “mentalist” while he was performing a telepathic task in a 1.5 T scanner. A matched control subject without this special ability was also examined under similar conditions.

**Results:**

The mentalist demonstrated significant activation of the right parahippocampal gyrus after successful performance of a telepathic task. The comparison subject, who did not show any telepathic ability, demonstrated significant activation of the left inferior frontal gyrus.

**Conclusions:**

The findings of this study are suggestive of a limbic basis for telepathy and warrant further systematic research.

## INTRODUCTION

“Telepathy” is defined as “the communication of impressions of any kind from one mind to another, independently of the recognized channels of sense”.[1] With the help of various rigorous paradigms over the last 70 years, systematic research has lent support to the reality of telepathy.[2] Meta-analyses of “ganzfield” studies[3] as well as “card-guessing task”[4] studies provide compelling evidence for the existence of telepathy. This mysterious phenomenon has implications not only in the cognitive sciences but also in the biological and healing sciences.[2] It has long been assumed that conscious intention has the capacity to affect living systems across a distance. Intercessory prayers, healing energy, and similar other methods have long been a part of medicine.[2] Hence, analyzing the underpinnings of telepathy might potentially help in understanding the “distant-healing” phenomena also.

Examining people with extraordinary capabilities involving paranormal phenomena might help in a better understanding of these puzzling entities.[5] Previous such studies examining people with “special talents”[5,6] yielded significant insights. Similarly, studies have been conducted on people experiencing paranormal phenomena. A functional MRI study on “distant intentionality” (defined as sending thoughts at a distance) examined the brain activation pattern in a recipient of thoughts from healers who espoused some form for connecting or healing at a distance. The recipient demonstrated significant brain activations in the anterior and middle cingulate areas, precuneus, and the frontal regions.[7] Previous studies[8,9] examining subjects with telepathic ability suggested an association of paranormal phenomena with the right cerebral hemisphere. It has been reported that correlated neural signals may be detected by fMRI in the brains of subjects who are physically and sensorily isolated from each other.[10] In light of these previous studies, we aimed to examine the functional neuroanatomical correlates of telepathy in Mr. Gerard Senehi, an “expert with telepathic ability (mentalist)” using functional Magnetic Resonance Imaging (fMRI).

## MATERIALS AND METHODS

### Subjects

Mr. Gerard Senehi [Mr. GS] (aged 46 years) is well known for his abilities to perform various paranormal tasks such as telekinesis, mind reading, and telepathy (http://www.experimentalist.com). Mr. JS, the comparison subject, is a 43 –year-old male, who was aware of various paranormal phenomena including telepathy, but did not have any paranormal abilities to the best of his knowledge. Both the subjects were right-handed[11] and possessed Master's Degrees. Both the subjects were screened using the General Health Questionnaire[12] and a comprehensive mental status examination was done to rule out any psychiatric disorder. Neither of them had any history suggestive of substance abuse or dependence, medical or neurological disorders. Neither had any contraindication for MRI. The study procedures were explained to the subjects and written informed consent was obtained. The study protocol was reviewed and approved by the institute's ethics committee.

### Telepathy task

One of the investigators (PNJ) drew an image in the presence of other investigators [HRN, BNG, and GVS]. Figures 1A and 2A were the images drawn by PNJ for the “mentalist” and the control subject while both were seated in separate rooms. Neither the mentalist [GS] nor the control subject [JS] knew what the image was. The subject was then shifted to the MRI scanner and the investigator (PNJ) was seated in the MRI console room (about 15 feet away). Adequate precautions were taken to avoid sensory leakages by following the guidelines of Hyman and Honorton.[13] During the scan, the subject was instructed to perform the act of telepathy to think about and identify the probable image that would have been drawn by the investigator during the designated epochs of “activation” and not to engage in this task during the periods of “rest”. The subjects were visually cued (using a mirror attached to the head coil which reflected the cues projected on a screen) by green and red stars to indicate the respective onset of activation and rest epochs. The investigator (PNJ) was also given the same cues and was engaged in transmitting the image to the subject in the MRI scanner during the “activation” periods, stopping during the periods of rest. After the scanning, the subject was asked to draw the image that he was able to obtain by performing telepathy. Figure 1B was the image reproduced by the “mentalist” and Figure 2B was the image reproduced by the control. Both the subjects were scanned on the 3^rd^ day of the lunar cycle and at the same time of the day (1400 hours IST) separated by a three-month interval.

**Figure 1A:**
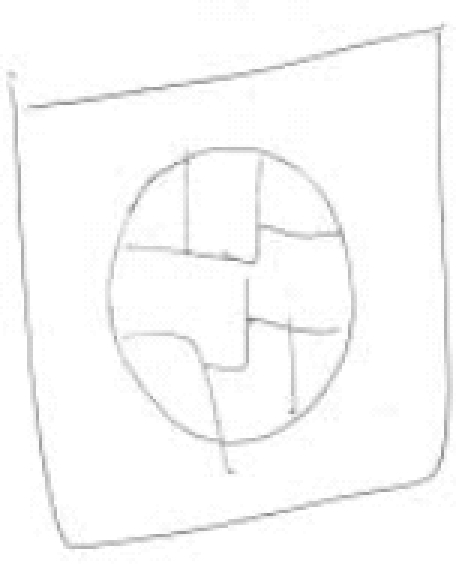
Image drawn by the investigator (PNJ) for the “mentalist” [Mr. GS]

**Figure 1B:**
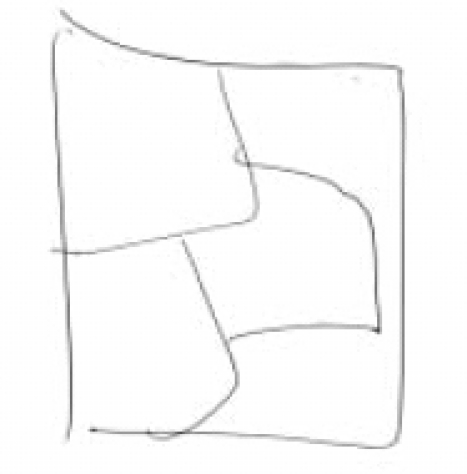
Image reproduced by the mentalist [Mr. GS] after the telepathic task

**Figure 2A:**
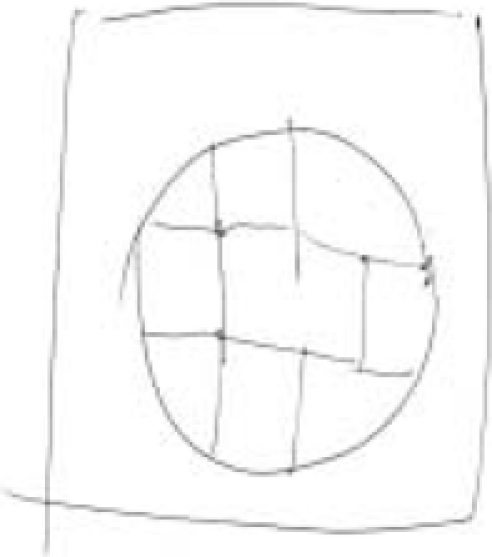
Image drawn by the investigator (PNJ) for the control subject [Mr. JS]

**Figure 2B:**
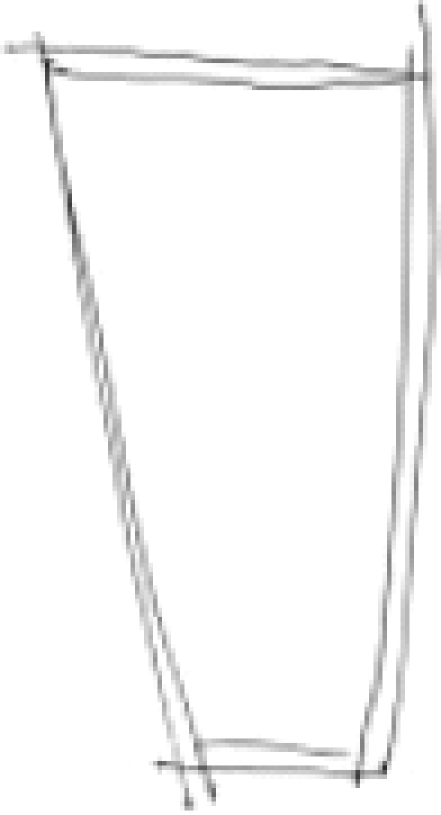
Image reproduced by the control subject [Mr. JS] after the telepathic task

### Imaging procedures

MRI was done with 1.5 Tesla Magnetom ‘vision’ scanner. First, a T_1_ -weighted three-dimensional Magnetization Prepared Rapid Acquisition Gradient Echo sequence was performed (TR = 9.7 msec; TE = 4 msec; nutation angle = 12°; FOV = 250 mm; slice thickness 1 mm; NEX = 1; matrix = 200 × 256; 160 sagittal slices). After obtaining the anatomical MR images, echo-planar images (EPI) were obtained. They consisted of 112 functional acquisitions, with each acquisition consisting of 16 slices (slice thickness = 8 mm without any interslice gap) in the axial plane covering the entire brain. The parameters for a multishot EPI sequence using Blood Oxygen Level Dependent (BOLD) contrast were as follows: repetition time = 4000 msec; echo time = 76 msec; flip angle = 90°; FOV = 250 mm; matrix 128 × 128. The acquisitions were grouped in blocks of eight, yielding 14 blocks. The condition for successive blocks alternated between “rest” and the “telepathic” task, starting with “rest”. This “rest-telepathy” paradigm yielded seven sets of “rest” and “telepathy”.

### Image analysis

The fMRI analysis was performed using Statistical Parametric Mapping-2 (SPM2)(http://www.fil.ion.ucl.ac.uk/spm). The EPI images were realigned and corrected for slice timing variations. The images were then normalized[14] to the Montreal Neurological Institute (MNI) space.[15] Finally, the images were smoothened with a gaussian kernel of 6 mm full-width, half-maximum.

SPM2 combines the General Linear Model and Gaussian field theory to draw statistical inferences from BOLD response data regarding deviations from the null hypothesis in three-dimensional brain space.[16] The images were analyzed using a block design paradigm with a canonical hemodynamic response function. The epochs of rest were subtracted from the epochs of the telepathic task performance. The voxel-wise analysis produced a statistical parametric map of brain activation associated with the telepathic task in the MNI space. Significance corrections for multiple comparisons were performed using a False Discovery Rate (FDR) correction[17] (*P* < 0.05). The coordinates of significant areas of activation were transformed from MNI space[15] into the stereotactic space of Talairach and Tournoux[18] using nonlinear transform.[19] The brain regions were localized from the Talairach and Tournoux co-ordinates using automated software.[20]

## RESULTS

**Figure 3:**
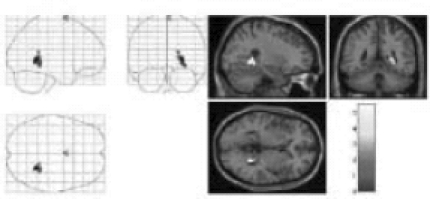
Right Parahippocampal Gyrus Activation in the subject with telepathic ability [Mr. GS], while performing a successful telepathic task. On the left hand side, the activation is superimposed on a glass brain and on the right hand side, the activation [yellow] is superimposed on a structural MR image

**Figure 4:**
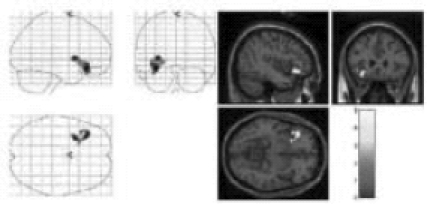
Left Inferior Frontal Gyrus Activation in the control subject without any telepathic ability [Mr. JS], while performing an unsuccessful telepathic task. On the left hand side, the activation is superimposed on a glass brain and on the right hand side, the activation [yellow] is superimposed on a structural MR image

The image [Figure 1B] reproduced by the “mentalist” showed striking similarity to the original image drawn by the investigator (PNJ) whereas the one reproduced by the control subject [Figure 2B] did not. The mentalist showed significant activation involving the right parahippocampal gyrus [Number of voxels = 160; Talairach and Tournoux co-ordinates of peak activation: ‘x’ = 32, ‘y’ = -41, ‘z’ = -6; T = 4.88; *P* (uncorrected) < 0.001; FDR-corrected *P* = 0.018] [Figure 3] whereas the control subject showed significant activation involving the left inferior frontal gyrus [number of voxels = 363; Talairach and Tournoux co-ordinates of peak activation: ‘x’ = -42, ‘y’ = 25, ‘z’ = -8; T = 4.21; *P* (uncorrected) < 0.001; FDR-corrected *P* = 0.037] [Figure 4].

## DISCUSSION

To our knowledge, this is the first fMRI study to examine the brain correlates of telepathy. Previous studies have employed other functional brain mapping techniques such as Single Photon Emission Computed Tomography (SPECT)[8] and electroencephalography (EEG) and MRI[9] to investigate paranormal phenomena in selected individuals. In our study, telepathy was associated with significant activation of the right parahippocampal gyrus; whereas the control subject without telepathic ability, activated the left inferior frontal gyrus under similar task conditions.

A previous study[9] on Mr. Ingo Swann (who had the special ability of remote-viewing) showed that the proportions of unusual 7-Hz EEG spike and slow wave activity over the occipital lobes per trial had a correlation with the ratings of response accuracy. Neuropsychological and MRI analyses suggested a differential structural and functional organization within the parieto-occipital region of Mr. Swann's right hemisphere.

Another SPECT study[8] examined Mr. Sean Harribance, who routinely experienced “flashes of images” of objects that were hidden and of accurate personal information concerning people with whom he was not familiar. The “extrasensory” processes in Mr. Harribance correlated quantitatively with morphological and functional changes involving the right parietotemporal cortices (or its thalamic inputs) and hippocampal formation.

Together, these two studies suggest that paranormal phenomena might have a relationship with the right cerebral hemisphere, especially the right posterior cortical and hippocampal regions. The parahippocampal region is very closely linked to the hippocampus, both structurally and functionally.[21] So, the current study findings also support the association between the right hippocampal system and paranormal phenomena.

In our study, the control subject activated his left inferior frontal gyrus during his unsuccessful telepathic task performance; this brain area is implicated in the “Theory of Mind [ToM]”.[22] The attribution of mental states, such as desires, intentions, and beliefs, to others has been referred to as ToM.[23] Empathy, conceptually related to ToM, is described as the ability to infer and share the emotional experiences of another.[24] An earlier study reported that psychic mind readers had greater cognitive empathy than individuals without these abilities.[5] Importantly, hippocampal brain regions are important for empathy.[25] Thus, our observations derive indirect support from this earlier study.[5]

Superior empathizing abilities have been hypothesized to be important for both telepathy[5] as well as for distant intentionality.[7] Interestingly, the cuneus (a brain region associated with empathy[26]) has been reported to be linked with distant intentionality.[7] Also, in our study, the hippocampal region (associated with empathy[25]) is implicated in telepathy. These observations support the hypothesized link between empathy and special abilities. It is possible that people with telepathy or distant healing abilities might possess the ability to activate differentially specific brain regions (in localization, *e.g*, anterior *vs* posterior brain regions or in lateralization, *e.g*, right *vs* left brain) related to the empathy circuit in comparison to individuals without these abilities.

On the contrary, empathy deficits[27] and cuneus[28] and parahippocampal abnormalities[29] and anomalous right hemisphere overactivation[30] have been reported in schizophrenia. Most of these “left-hemisphere dominance failure” findings have been conceptualized as being “abnormal” in their tendency to increase a person's proclivity towards psychosis. Paradoxically, evolutionary theories on psychosis propose an alternative possibility that some of these traits might be of crucial utility.[31] It has been proposed that this dominance failure (and consequent right hemisphere overactivation) facilitates the emergence of paranormal and delusion-like ideas by way of right hemispheric associative processing characteristics, *i.e.*, coarse rather than focused semantic activation. Interestingly, the ability to detect subtle magnetic field energies might underlie paranormal phenomena.[32] Moreover, magnetic field abnormalities have been described to be the underlying basis for psychotic symptoms.[33,34] However, it is yet to be examined whether a conglomeration of these features (*i.e.*, reduced left hemispheric dominance, paranormal beliefs) are also indicative of an inherent advantage towards acquiring “special” abilities in some people (of course, with enhancement towards psychosis in others) possibly due to an enhanced tendency to perceive subtle geomagnetic energy alterations.

Ours is probably the first fMRI study to examine the neuroanatomical correlates of telepathy. fMRI offers methodological advantages of nonradioactive and noninvasive real-time imaging of the brain. We have employed a well-researched and validated image analysis paradigm with optimal correction for false positive results. Our study methodology strictly adhered to the guidelines for research on paranormal phenomena proposed by Hyman and Honorton.[11] These include rigorous precautions against sensory leakage, extensive security procedures to prevent malpractices, full documentation of all experimental procedures and equipment, and complete specifications about statistical analyses.

Nonetheless, one has to be cautious while interpreting the study findings due to the following limitations: i) ideally, it would have been methodologically more rigorous if Mr. Gerard had replicated the successful telepathic task with similar brain activation during another session of fMRI on a different occasion. As Mr. Gerard had reported some inexplicable discomfort in the few days following the fMRI, this could not be done; Ii) examination of just one control subject is another limiting factor.

## CONCLUSIONS

In summary, this study's findings are suggestive of an association between telepathy and the right parahippocampal gyrus. The methodological rigor, isolated and robust brain activation with telepathy, and established theoretical relevance of this brain region with reference to paranormal phenomena highlight the need for further studies using advanced fusion imaging techniques (simultaneous fMRI, EEG, and magnetoencephalography) to examine telepathy.
